# *De novo* 8p21.3→ p23.3 Duplication With t(4;8)(q35;p21.3) Translocation Associated With Mental Retardation, Autism Spectrum Disorder, and Congenital Heart Defects: Case Report With Literature Review

**DOI:** 10.3389/fped.2020.00375

**Published:** 2020-07-08

**Authors:** Cristina Gug, Dorina Stoicanescu, Ioana Mozos, Laura Nussbaum, Mariana Cevei, Danae Stambouli, Anca Gabriela Pavel, Gabriela Doros

**Affiliations:** ^1^Department of Microscopic Morphology, Victor Babes University of Medicine and Pharmacy, Timisoara, Romania; ^2^Department of Functional Sciences, Victor Babes University of Medicine and Pharmacy, Timisoara, Romania; ^3^Center for Translational Research and Systems Medicine, Victor Babes University of Medicine and Pharmacy, Timisoara, Romania; ^4^Department of Neurosciences, Victor Babes University of Medicine and Pharmacy, Timisoara, Romania; ^5^Department of Psychoneuro Sciences and Rehabilitation, Faculty of Medicine and Pharmacy, University of Oradea, Oradea, Romania; ^6^Department of Molecular Genetics and Cytogenetics, Cytogenomic Medical Laboratory, Bucharest, Romania; ^7^Department of Pediatrics, Victor Babes University of Medicine and Pharmacy, Timisoara, Romania

**Keywords:** 8p(21.3–p23.3) duplication, translocation(4;8), *de novo*, array CGH, FISH, mental retardation, autism spectrum disorder, congenital heart defects

## Abstract

Duplications of chromosome 8p lead to rare genetic conditions characterized by variable phenotypes. 8p21 and 8p23 duplications were associated with mental retardation but only 8p23 duplication was associated with heart defects. 8p22→ p21.3 duplications were associated with an autism spectrum disorder in several cases. We present a rare case with a *de novo* duplication of the entire 8p21.3→ p23.3 region, documented by karyotype, FISH, and array CGH, with t(4;8)(q35;p21.3) translocation in a 7 years-old girl. She was referred for genetic counseling at the age of 20 months due to mild dysmorphic facial features, psychomotor retardation, and a noncyanotic heart defect. Another examination carried out at the age of 5 years, enabled the diagnosis of autism spectrum disorder and attention deficit hyperactivity disorder. Upon re-examination after two years she was diagnosed with autism spectrum disorder, attention deficit hyperactivity disorder, liminal intellect with cognitive disharmony, delay in psychomotor acquisitions, developmental language delay, an instrumental disorder, and motor coordination disorder. Cytogenetic analysis using GTG technique revealed the following karyotype: 46,XX,der(4),t(4;8)(q35;p21.3). The translocation of the duplicated 8pter region to the telomeric region 4q was confirmed by FISH analysis (DJ580L5 probe). Array CGH showed: arr[GRCh37]8p23.3p21.3(125733_22400607) × 3. It identified a terminal duplication, a 22.3 Mb copy number gain of chromosome 8p23.3–p21.3, between 125,733 and 22,400,607. In this case, there is a *de novo* duplication of a large chromosomal segment, which was translocated to chromosome 4q. Our report provides additional data regarding neuropsychiatric features in chromosome 8p duplication. The phenotypic consequences in our patient allow clinical-cytogenetic correlations and may also reveal candidate genes for the phenotypic features.

## Introduction

Significance of segmental duplications leading to abnormal gene dosage and consequently to different disorders has been intensively studied in recent years (1).

Duplications of the short arm of chromosome 8 are structural chromosomal abnormalities that lead to rare genetic conditions characterized by a variable phenotype. Depending on the size and part of the chromosome that is duplicated, some individuals may be apparently normal, while others may have a range of clinical features from mild to very severe (2, 3). The duplicated material from 8p may remain on the same chromosome or, rarer, can be translocated to a different chromosome. It seems that the phenotypic effects are not influenced by the position of the duplicated part (4, 5). Some chromosome 8p duplications may be familial, but an appreciable number of cases result from *de novo* mutations (6–9). Although inverted duplications adjacent to terminal deletions of chromosome 8p are the most common, cases with direct duplications have been also described (10, 11).

### Known and Novel Scientific Information

- 8p21→ 8p23 duplications were associated with mental retardation (MR), interstitial duplication 8p23 was associated with congenital heart defects (CHDs).
- 8p23.1 duplication syndrome defined as a 3.75 Mb duplication of most band 8p23.1 is characterized by mild to moderate developmental delay, mental retardation (MR), mild facial dysmorphism, and CHDs.
- Duplications in chromosome 8p21.3→ p22 were previously associated with autism spectrum disorder (ASD).

We present a *de novo* duplication of the entire region 8p21.3→ p23.3, documented by karyotype, FISH, and array CGH, with translocation t(4;8)(q35;p21.3) in a 7 years-old girl with liminal intellect with cognitive disharmony, ASD, attention deficit hyperactivity disorder (ADHD) and cardiac defects.

- In this case, there is a *de novo* duplication of a large chromosomal segment, the duplicate part being translocated to chromosome 4q, without deletion of the 4q35.2 telomere, which has not been reported before.
- The phenotypic consequences in our patient allow clinical-cytogenetic correlations and may reveal candidate genes for the phenotypic features. Our case has a large duplication of the entire 8p21.3→ p23.1 region and displays a combination of clinical features: MR, ASD, ADHD, CHD, previously described separately for different duplicated chromosomal segments.
- The 8p23.1 region was associated with facial dysmorphism, especially prominent forehead, an important sign, noticed during infancy, that could guide investigations toward the identification of 8p duplication.
- We hypothesized that our patient's phenotype was entirely determined by the large duplication of 8p21.3→ p23.1 region.

## Clinical Presentation and Family History

We report the case of a 7-year-old female patient, referred for genetic counseling due to dysmorphic features, psychomotor retardation, and noncyanotic CHD. She was the first child of an apparently healthy, non-consanguineous couple. Both parents were 28 years old when the patient was born. Pregnancy was uneventful; the mother denied any exposure to alcohol, radiation, or infectious agents. Family history was negative for mental retardation and congenital defects and the karyotype of the parents was normal. However, the mother had two miscarriages before the proband was born. The patient has two healthy younger sisters, the prenatal diagnosis was performed during both pregnancies.

### Medical History

Clinical examination at birth revealed systolic murmur gr 3/6, heard over second, third, and fourth intercostal spaces at the left sternal border. Echocardiography performed 1 month later revealed noncyanotic CHD with ventricular septal defect (VSD) and patent foramen ovale. Developmental milestones acquisition delay was noticed at 20 months. There was also a delayed acquisition of language milestones; the patient had not verbally spoken a word. Nonetheless, nonverbal communication was relatively good, she smiled, but could not concentrate, did not establish visual contact, and did not respond when her name was called. She was referred for genetic counseling at this age due to mild dysmorphic facial features (prominent forehead, flat nasal bridge, low-set ears), psychomotor retardation, and noncyanotic CHD. aCGH was performed and showed a 22.3Mb duplication of the 8p21.3→ p22 region. The karyotype revealed the translocation of the duplicate fragment on chromosome 4q, terminal. The FISH analysis confirmed the duplication and translocation and showed that the 4q telomeres were preserved. At 2 years and 10 months, the first psychological assessment was performed. Low or average levels of all skills (cognitive, emotional, social, personal autonomy) were recorded. At 3 years and 9 months Early Childhood Inventory-4 screening used to assess common symptoms of psychiatric disorders in children aged 3–7 years, indicated ADHD characterized by hyperactive and impulsive inattentive behavior, in which attention deficit with medium-high severity prevailed. Other findings were physical and symbolic aggression, with specific phobia, selective mutism, and elimination disorders. The psychological evaluation using the Portage-Scale indicated that at the chronological age of 4 years and 8 months she had a mental age of 3 years and 9 months and DQ = 81 (developmental quotient). The pediatric neuropsychiatrist monitored the evolution, establishing mild mental retardation at the age of 5 years (IQ = 75 BS; Binet-Simon Intelligence scale), ASD, and ADHD.

## Methodology

### Clinical Evaluation

The patient underwent a multidisciplinary evaluation, involving health professionals from the following specialities: genetics, cardiology, pediatrics, pediatric neuropsychiatry, speech pathology, and psychology.

The patient's parents gave written informed consent (including for publication of images) considering the Declaration of Helsinki. This study was approved by the Ethics Committee for Scientific Research of the Emergency Hospital for Children “Louis Turcanu,” Timisoara.

### Neuropsychiatric Evaluation

The neuropsychiatric diagnoses were put through clinical examination, assessment of the diagnostic criteria after the Diagnostic and Statistical Manual of Mental Disorders (DSM5) and confirmed through Kiddie Schedule for Affective Disorders and Schizophrenia (K-SADS). The patient was also evaluated considering the Childhood Autism Rating Scale (CARS), Autism Diagnostic Interview-Revised (ADI-R), Autism Diagnostic Observation Schedule (ADOS), the Portage-Scale and ADHD-Rating Scale.

### Peripheral Blood Karyotype

Peripheral blood lymphocytes were cultured in a growth medium (PB-MAX™ Karyotyping Medium, Gibco). Metaphase chromosomes were harvested, and slides were made for analysis. GTG banding was used for staining (at the 550-band level). Chromosomal analysis was performed using LUCIA Karyo-G software, and the aberrations and karyotypes were classified according to the ISCN 2016 system.

### Fluorescence *in situ* Hybridization (FISH)

Aquarius ®Cytocell FISH probes were applied to metaphases. The probes were: (a) Subtelomere Specific Probes 8p (DJ580L5-red) and (d) 8q (489D14-green), (b) Wolf-Hirschhorn Syndrome Critical Region (WHSCR) Probe with Subtelomere Specific Probe red, which corresponds to 4p16.3, and (DJ963k6-green), which corresponds to 4q35.2, (c) alpha-satellite 8 probe (D8Z2-green) and Whole Chromosome Painting Probe (wcp4-red).

Array Comparative Genomic Hybridization (aCGH) analysis was performed using CytoChip Focus Constitutional BAC array (Illumina Inc., U.S.) and the used reference was the normal human male genomic DNA (Promega, WI). The DNA probes were derived from BAC (Bacterial Artificial Chromosomes) DNA clones from human genome collection. The selected BACs were replicated to a high degree, 3 × and 4 ×, providing a more robust signal for each data point. The target solution was hybridized to the 2 × 180K array and data were acquired using the InnoScan 710 microarray scanner. A composite image was obtained and imported into the Blue Fuse Multi Software (v.3.1) microarray software, Genome Assembly NCBI Build 37hg19, for data analysis. The average whole-genome resolution was approximately 1Mb. The DGV (Database Genomic Variation), Decipher (Database of Chromosomal Imbalance and Phenotype in Human using Ensembl Resources), ISCA (International Standards for Cytogenomic Arrays), OMIM (Online Mendelian Inherited in Man) international databases were used to interpret the results.

## Results

**Figure 1:**
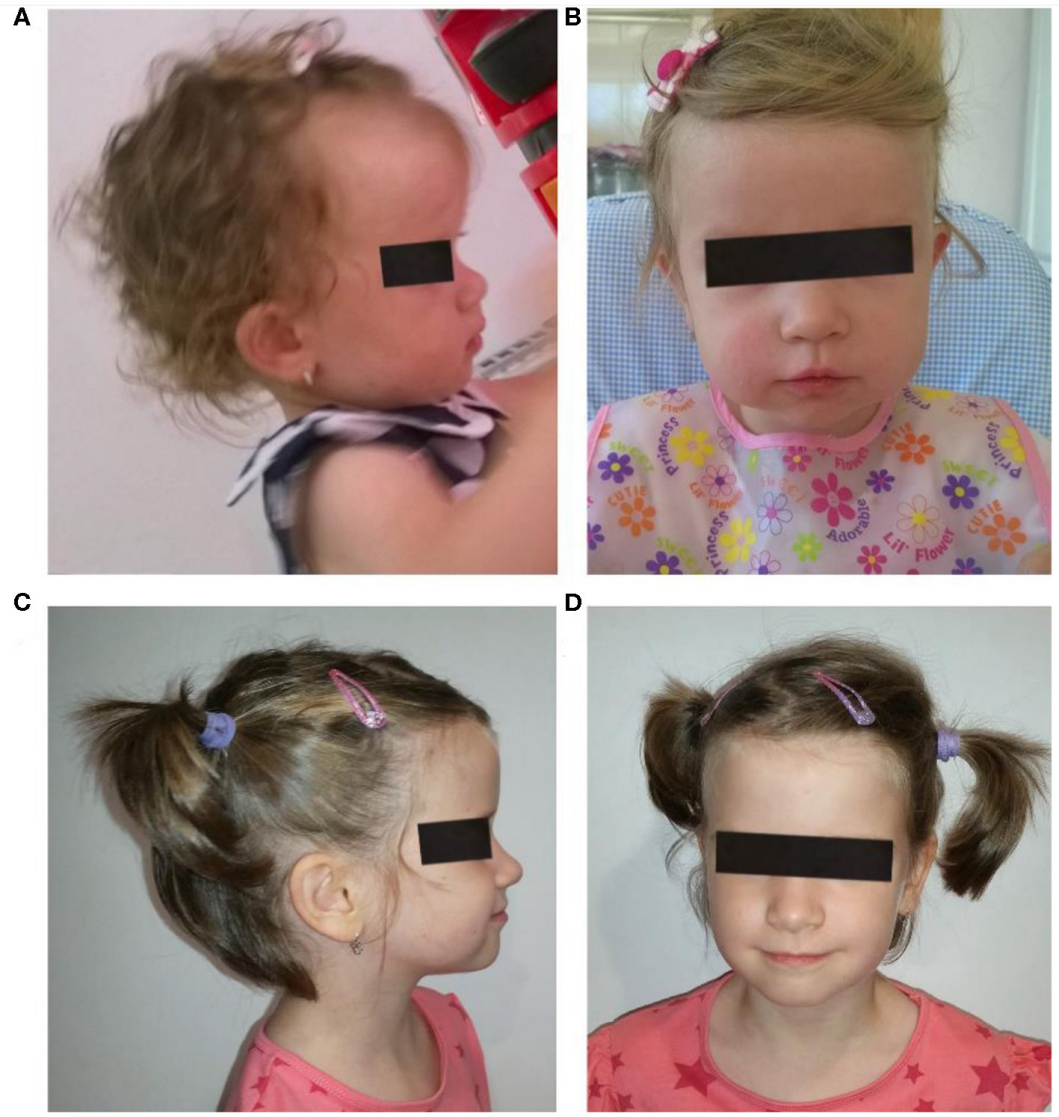
Patient face and profile at the age of 1 year **(A,B)** and 6 years **(C,D)**. Mild facial dysmorphism with prominent forehead, flattened nose base, and low set ears can be noticed.

Clinical evaluation at the ages of 1 and 5 years showed mild dysmorphic facial features: prominent forehead, flat nasal bridge, and diastema (Figure 1). In the differential diagnosis of the prominent forehead, the most striking dysmorphic feature, Crouzon syndrome, Hurler syndrome, Pfeiffer syndrome, Rubinstein–Taybi syndrome, and Russell–Silver syndrome have been considered. They were ruled out due to the absence of other specific clinical features.

At the age of 6 years and 11 months, she was evaluated by the pediatric neuropsychiatrist, her diagnosis being ASD, ADHD, delay in psychomotor acquisitions with mild delay in the expressive language acquisition with polymorphic dyslalia, motor coordination disorder and instrumental disorders, liminal intellect with cognitive disharmony (IQ = 83 WISC; Wechsler Intelligence Scale for Children), minimal brain lesions indices, organic brain background. Related to the diagnosis of ASD, the patient showed qualitative deterioration of social interaction and cognition, communication, lack of emotional reciprocity and empathy, no make-believe play, lack of imaginative play, and also stereotyped, repetitive behavioral patterns and mannerisms and also resistance to change.

In the first stage of the differential diagnosis for ASD, we excluded organic disorders, which could show an “autistic-like” behavior, through the neurological examination and paraclinical investigations. In the second stage, we made an accurate differential diagnosis with all the psychiatric disorders, revealing an “autistic-like” behavior–mental retardation, language development disorders, ADHD, sensory disorders, reactive attachment disorders, early-onset schizophrenia. The clinical evaluation, the psychiatric exam, the psychological evaluations, and the applied scales guided us.

We also made a differential diagnosis of ADHD, ruling out other medical and psychiatric disorders with cognitive, attentional and executive functioning deficits and hyperkinetic impulsive behavior or agitation, restlessness–anxiety disorders, attachment disorders, posttraumatic stress disorder, and mood disorders, including depressive or bipolar disorders. In children, ADHD and bipolar disorders can have overlapping symptoms. Both can present with distractibility, increased energy, and mood lability, and, therefore, a thorough history is essential for the diagnosis.

The prognosis is guarded and symptomatic psychopharmacologic treatment, psychological therapies, psycho-sensorial stimulation, preschool special education, occupational, physical, speech, developmental, and behavioral therapies were helpful for the management of this complex case. Antipsychotic, mood-stabilizing, and neurotrophic medication but also behavioral therapies, like ABA (Applied Behavioral Analysis), PECS (Picture Exchange Communication Systems), or TEACCH (Treatment and Education for Autistic and Communication Handicapped Children) were recommended and might improve the prognosis and the clinical evolution of the patient.

Echocardiography revealed noncyanotic CHD with restrictive perimembranous DSV with a left to right shunt, covered by excess tissue from the tricuspid valve, patent foramen ovale, and bicuspid aortic valve with gr I aortic insufficiency. Cardiological monitoring was recommended and performance sports were contraindicated.

**Figure 2:**
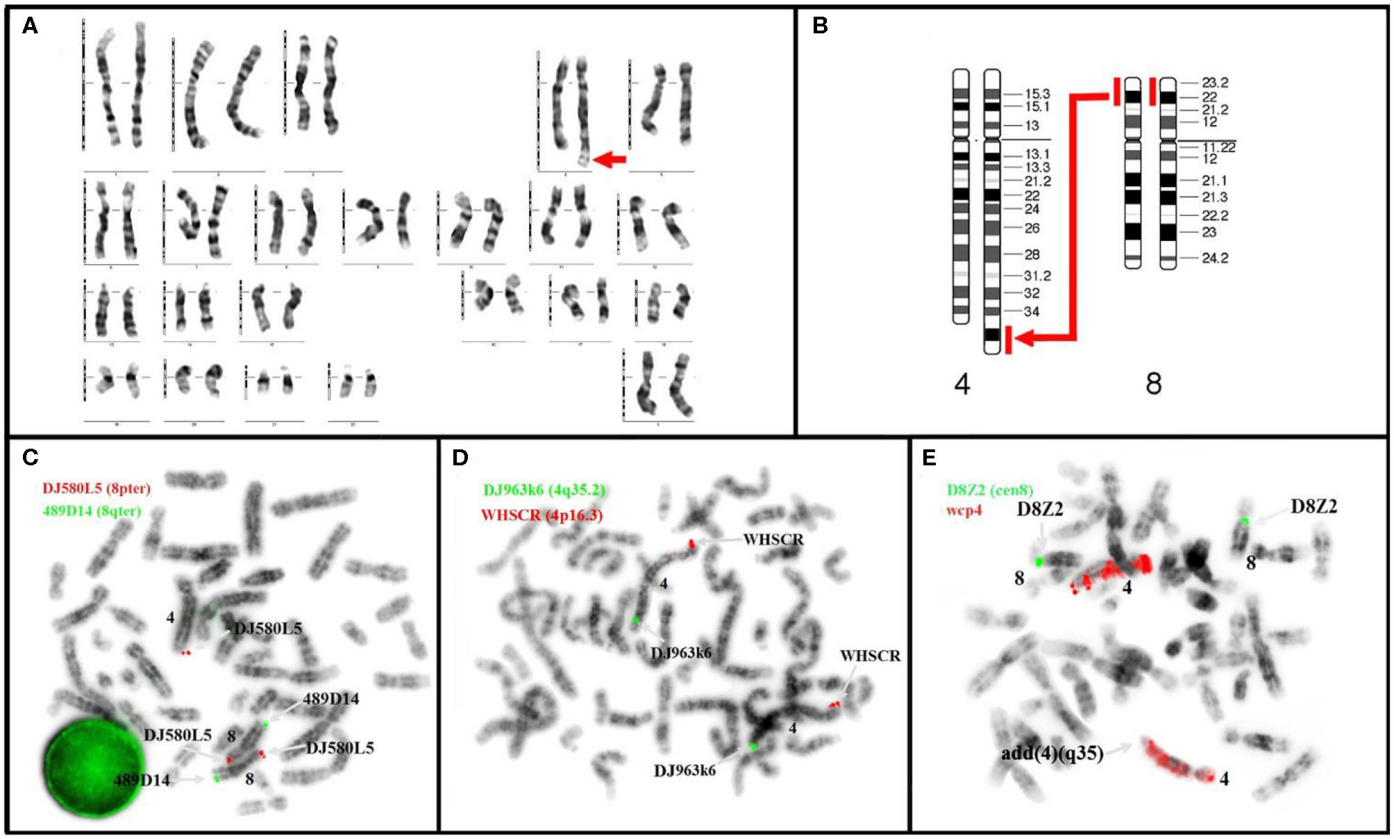
Karyotype **(A,B)** and metaphase FISH **(C,D)** of our patient revealed: **(A)** *de novo* duplication (p21.3→ p23.3) with translocation t(4;8)(q35;p21.3) (arrow); **(B)** diagram of partial trisomy 8p with the large arrow indicating the location of the duplicate segment; **(C)** Kit Aquarius® Specific Probes Red (DJ580L5) mark subtelomeric 8p and Green (489D14) mark subtelomeric 8q; 3 copies are noticed for 8p of which 2 correctly positioned and the third translocated to the telomeres of chromosome 4q, ish der(4)dup(4)t(4;8)(489D14+,DJ580L5++) **(D)** Kit Aquarius® Specific Probes Red (WHSCR) mark subtelomeric 4p16.3 and Green (DJ963k6) mark subtelomeric 4q35.2; the image prove s the presence of 4q telomeres: ish der(4)t(4;8)(WHSCR+,DJ963k6+). **(E)** Kit Aquarius® Whole Chromosome Painting Probes wcp 4 Red and Kit Aquarius® Satellite Enumeration Probes α-satellite 8 (D8Z2).

Cytogenetic analysis of the proband revealed a female karyotype with derivative chromosome 4. The following karyotype was revealed: 46,XX,der(4),t(4;8)(q35;p21.3). Hence, a partial trisomy of chromosome 8p, from band p21.3 to p23.3, resulted (Figures 2A,B). The duplicated fragment was translocated to chromosome 4q, terminal. The abnormality was found in all metaphases. The karyotype of the parents was normal; therefore, the abnormality is *de novo*.

FISH result was 46,XX,der(4),t(4;8)(q35;p21.3).ish der(4),t(4;8)(q35;p21.3)(wcp4+,WHSCR+,DJ963k6+,DJ580L5+). The duplication of the 8pter region and translocation to the telomeric region 4q were confirmed by FISH analysis (DJ580L5 probe) (Figures 2C,D).

**Figure 3:**
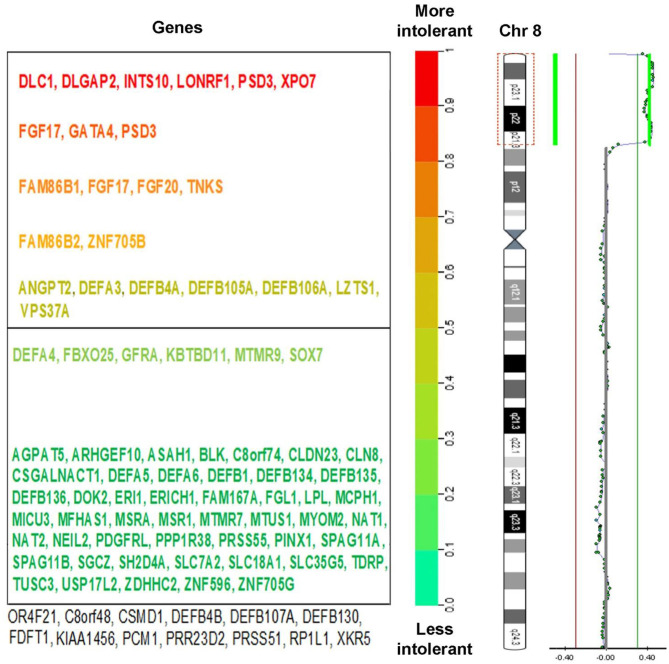
**(Right)** Chromosome view of a 22.3 Mb duplication of the 8p23.3-21.3 bands, arr[GRCh37] 8p23.3p21 (125733_22400607) × 3 detected by aCGH analysis and ideogram of chromosome 8. **(Left)** The genes within this region were marked with different colors, depending on their intolerance to mutations. Known pathogenic genes are marked in red (according to DECIPHER v9.31 database).

Array CGH showed: arr[GRCh37] 8p23.3p21.3(125733_22400607) × 3. It identified a terminal duplication, a 22.3 Mb copy number gain of chromosome 8p23.3–p21.3, between 125,733 and 22,400,607. Duplication limits using array platforms are illustrated in Figure 3. In the cytogenetic location delimited by the genomic coordinates (GRCh37-Genome Reference Consortium Human Build 37), there are 134 genes, located as follows: 1 gene in band 8p21; 30 genes in band 8p21.3; 39 genes in band 8p22; 57 genes in band 8p23.1; and 6 genes in band 8p23.3 (12).

## Discussion

Chromosome 8p duplication is a rare chromosomal aberration with unknown prevalence. Phenotypic features may be nonspecific and a combination of complementary tests that include karyotype, FISH analysis, and microarray are required for diagnosis. Table 1 summarizes the main information about 8p21.3→ p23.3 microduplication syndrome. The present study shows a rare case with a *de novo* duplication of the entire region 8p21.3–p23.3, which has not been reported before.

**Table 1:** 8p21.3→ p23.3 Duplication syndrome (in chronological order of the reports).

**Region with** **duplication**	**Size Mb**	**Type of** **duplication**	**±Translocation**	**Pattern**	**Abnormality**	**References**
8p21.3→ p23.3	Unknown	Duplication	der(12), t(8;12)(p12;pl3)	Familial 3 cases	MR, simian crease, CHD (atrial septal defect, ventricular septal defect)	(13)
8p12→ 8p21.1	Unknown	Direct duplication	No	Familial (3 cases)	mild MR	(6)
8p21.3→ 23.1	Unknown	Direct duplication in 7 cases	No	Familial (6 cases) sporadic (1 case)	Normal to moderate MR in the affected individuals, ADHD (1 case), CHD (2 cases including 1 prenatal)	(5)
8p21→ p23	Unknown	Duplication	No	*De novo*	ASD, mild dysmorphic features, and moderate learning disability, MR	(14)
8p23.1	3.75 Mb	Duplication	No	Sporadic	prominent forehead, mildly arched eyebrows, slightly upward slanting palpebral fissures	(15)
8p21→ 8p23.1	12 Mb	Direct duplication 8p + deletion 8p23.1	Rearrangement 8p with der(8)dirdup(8)(p21p23.1) del(8)(p23.1pter)	*De novo*	global developmental delays, seizures, Dandy–Walker variant	(10)
8p23.1→ 8p23.2	6.8 Mb	Duplication	No	Maternal	Child: speech delay, ASD, mother: epilepsy and learning problems	(16)
8p21	6.14 Mb	Duplication	no	*De novo*	Cognitive and motor development severely MR, facial dysmorphic features, ASD, self-mutilation	(17)
8p23.1.	Minimum 3.79 Mb	Interstitial duplication	no	4 cases including 2 familial (maternal)	CHD (aortic stenosis) prominent forehead	(18)
	Maximum 5.26 Mb	Terminal duplication				
	6.83 Mb	Terminal duplication	der(8)t(8;15)(p22;q24.1)	1 case	Prominent forehead CHD (aortic dilatation)	
8p23.1.	1.8 Mb	Interstitial duplication	no	Familial	Delay of motor and speech development, MR, ASD.	(19)
8p23.1.	3.68 Mb	Duplications	no	Familial	Developmental delay, dysmorphism including a prominent forehead and arched eyebrows., macrocephaly and, but not CHD	(20)
8p21.3→ p23.3	Unknown	Duplication	der(16), t(8;16)(p21;q24)	Maternal	CHD (transverse aortic arch hypoplasia)	(21)
8p21.3→ p23.3	22.3 Mb	8p terminal duplication	der(4),t(4;8)(q35;p21.3)	*De novo*	Proeminent forehead, mild MR, ASD, ADHD, noncyanotic CHD (ventricular septal defect)	Present case, 2020

*ADHD, Attention Deficit Hyperactivity Disorder; ASD, Autism Spectrum Disorder; CHD, congenital heart defect; der, derivative; Mb, megabase; MR, mental retardation; t, translocation*.

More than 50 cases with 8p inverted duplication/deletion syndrome have been described but, direct duplications of chromosome 8p are less common (5, 6, 11).

8p23.1 duplication syndrome defined as a 3.75 Mb duplication, most of band 8p23.1, has a prevalence of 1–9/100,000 (15, 22). The core 3.68 Mb duplication contains 32 genes of which five were considered candidates for the phenotypic features. Barber et al. (20) suggested that 8p23.1 duplication syndrome is an oligogenic condition, largely caused by the duplication and interactions of the SOX7 and GATA4 transcription factors. They noticed that eight genes led to developmental delay and dysmorphism including macrocephaly, prominent forehead, and arched eyebrows. Centromeric 8p23.1 microduplications including the GATA4 gene could enable the development of non-syndromic congenital heart defects (15, 20). An evaluation of 1645 pediatric patients with different developmental disorders by high-resolution microarray based CGH found four cases with a ≈4.0 Mb interstitial duplication of 8p23.1 (18). A patient with delayed motor and speech development and intellectual disability had a 1.80 Mb duplication in 8p23.1. SOX7 and TNKS1 genes and possibly MIR124-1 and MIR598, located within this interval, were probably responsible for the pathognomonic features of the syndrome (19).

Direct 8p21.3→ p23.3 duplication has been reported in one case with the following karyotype: 46,XX,der(16)t(8;16)(p21;q24)mat. Unlike our patient who had a *de novo* duplication, the other case resulted from a balanced maternal translocation (21).

The duplicated region in our case included 134 genes (12). The identified copy number variation was associated with 8p21.3→ p23.3 microduplication syndrome, being a genomic imbalance, described in international databases as having pathological significance. The duplicated region has an important gene content associated with the development of pathological conditions, particularly with growth retardation, dysmorphic features, skeletal abnormalities, which may predispose to restrictive lung disease and congenital heart defects (23, 24). Other phenotypic consequences are developmental delay, mental retardation, and behavioral problems. Our patient had facial dysmorphism, mild mental retardation/in dynamics liminal intellect with cognitive disharmony, delay in psychomotor acquisitions, developmental language delay, cognitive disharmony, instrumental and motor coordination disorder, ASD, and ADHD. The chromosomal segment 8p21.1→ p21.3 seems to be the critical region for 8p duplication syndrome (5). A mild clinical outcome for trisomy 8p22→ 8pter was reported in a study, in contrast to the severe findings when the duplication involved a longer, more proximal segment (25).

An 8p23.1→ 8p23.2 duplication spread over 6.8 Mb has been reported in a child with speech delay and autism and his mother, with epilepsy and learning problems. The interval included 41 known genes and 32 new genes among which the MCPH1 gene was thought to be the only plausible candidate gene for autism (16).

Papanikolaou et al. (14) described a patient with partial trisomy 8p(21-23) associated with autism, mild dysmorphic features, and moderate learning disability. Pinto et al. (26) suggested that the DLGAP2 gene, located on chromosome 8p23.3, could be a novel candidate gene for ASD. Further evidence for the DLGAP2 gene as a strong candidate gene has been provided by Poquet et al. (27) based on several cases with *de novo* duplications involving the DLGAP2 gene and presenting with ASD. Our patient also had a *de novo* duplication that included 8p23.3, DLGAP2 gene, respectively. Different chromosomal interstitial 8p rearrangements, including duplications, have been associated with ASD (17, 28, 29).

The size of the duplicated region correlated with the degree of cognitive deficiency in a patient with severe developmental and intellectual disability, severe impairment of expressive speech, and language (30).

CHDs were found only in a few patients with 8p23.1 duplication containing the GATA4 gene (18). These defects are different, even within the same family there were cases with aortic stenosis or aortic dilatation (18). Transverse aortic arch hypoplasia has been reported in two fetuses prenatally diagnosed with 8p23.3–p21.3 trisomy and with direct duplication of 8p21.3–p23.1, respectively (5, 21). Cardiac defects can manifest in unexpected forms in different syndromes, but they are always an important element in assessing complex cases (31).

Only extremely rarely, the 8p duplicate material is translocated to another chromosome. In our patient the cytogenetic analysis identified the translocation t(4;8)(q35;p21.3), therefore karyotyping remains a useful method to detect chromosomal rearrangements (32–34). The trisomic fragment 8p has been translocated to the end of 4q with the complete preservation of 4q telomeres. FISH analysis indicated preservation of telomeres; therefore, we hypothesize that our patient's phenotype is determined entirely by the large duplication of 8p21.3→ p23 region.

A search of PubMed using the keywords: “chromosome 8p duplication” and “translocation” revealed several reports referring to various chromosomal rearrangements involving chromosome 8. We excluded cases that had combined chromosome anomalies, and we found three reports of 8p duplication in offspring of carriers of balanced translocations involving chromosome 8. A report presented a family with t(8;12)(p12;pl3) carriers and affected descendants with 8p21.3→ p23.3 duplication, associated with MR and CHD (13). Translocation t(8;15)(p22;q24.1) and duplication 8p associated with CHD and prominent forehead in the offspring have been found in another family, as well. In the third family, a pregnant woman was a carrier of a balanced translocation t(8;16)(p21;q24), while the fetus had an unbalanced translocation 46,XX,der(16)t(8;16)(p21;q24)mat, associated with CHD (21).

Clinico-cytogenetic correlations revealed associations between phenotypic features and certain duplicate regions. Thus, 8p12 region was associated with MR (6), 8p23.1 region was associated with MR, ASD (16, 19), facial dysmorphism, especially prominent forehead (15, 18, 20), and CHD (18). 8p21.3→ p23.1 region was associated with MR (5, 13, 14, 17), ADHD (5, 17), ASD (27) and CHD (5, 13, 21). This study shows only one case that has a large duplication involving the entire 8p21.3→ p23.1 region, associated with mild facial dysmorphism with a prominent forehead, MR, ADHD, ASD, and CHD. This is preliminary evidence that indicates that all patient's symptoms are caused by this *de novo* duplication.

The origin of the duplicated segment has not been determined in our study. A report of 52 cases of *de novo* unbalanced translocations indicated that the primary driver for their occurrence was a maternal meiotic non-disjunction, followed by partial trisomy rescue (35).

A FISH analysis study found a direct tandem 8p duplication and, unlike inv dup del(8p), this was not derived from parental submicroscopic inversion (10). FISH method alone or in combination with other tests is highly informative (36). The phenotype of inverted duplications 8p is distinct from and much more severe than the clinical effect of partial trisomy 8p due to direct duplications known so far (6). Genetic counseling must consider that gonadal mosaicism cannot be excluded (37, 38). For this reason, prenatal diagnosis was performed in subsequent pregnancies (39–41).

## Conclusions

We present a mildly affected phenotype correlated with a *de novo* 22.3Mb copy number gain of chromosome 8p21.3–p23.3, in a patient with liminal intellect with cognitive disharmony, autism spectrum disorder, attention deficit hyperactivity disorder, delay in psychomotor acquisitions and a noncyanotic congenital heart defect. Our results suggest that our patient's phenotype can be explained by the large duplication of 8p21.3→ p23.1 region. Our report emphasizes the diagnostic value of molecular cytogenetics in children with an autism spectrum disorder. It also provides additional data regarding neuropsychiatric features in chromosome 8p duplication.

## Data Availability Statement

All datasets generated for this study are included in the article/supplementary material.

## Ethics Statement

The studies involving human participants were reviewed and approved by Ethics Committee for Scientific Research of Emergency Hospital for Children ‘Louis Turcanu,' Timisoara, Romania. Written informed consent to participate in this study was provided by the participants' legal guardian/next of kin. Written informed consent was obtained from the minor(s)' legal guardian/next of kin for the publication of any potentially identifiable images or data included in this article.

## Author Contributions

CG and DSto are the co-first authors. CG performed the cytogenetic analysis, the genetic counseling, and wrote the first draft of the manuscript. DSto coordinated and supervised data collection and interpretation, and critically reviewed the manuscript for important intellectual content. GD is the cardiologist who followed the child throughout the evaluation and made essential contributions to the manuscript writing. LN, MC, DSta, and AP collected the data, analyzed, and interpreted the findings, critically revised, and reviewed the manuscript for important intellectual content. IM revised and improved the first draft of the manuscript and is the corresponding author. All authors have read and approved the final manuscript for publication.

## Conflict of Interest

The authors declare that the research was conducted in the absence of any commercial or financial relationships that could be construed as a potential conflict of interest.

## Abbreviations

ABA: Applied Behavioral Analysis
aCGH: Array Comparative genomic hybridization
ADHD: Attention deficit hyperactivity disorder
ADI-R: Autism Diagnostic Interview-Revised
ADOS: Autism Diagnostic Observation Schedule
ASD: Autism spectrum disorder
BAC: Bacterial Artificial Chromosomes
BS: Binet-Simon Intelligence scale
CARS: Childhood Autism Rating Scale
CHD: Congenital heart defect
DGV: Database Genomic Variation
DSM-5: Diagnostic and Statistical Manual of Mental Disorders
GTG banding: Giemsa Trypsin G-band
IQ: Intelligence quotient
ISCA: International Standards for Cytogenomic Arrays
K-SADS: Kiddie Schedule for Affective Disorders and Schizophrenia
Mb: megabase
MR: mental retardation
PECS: Picture Exchange Communication Systems
t: translocation
TEACCH: Treatment and Education for Autistic and other Communication Handicapped Children
VSD: Ventricular septal defect
WAIS: Wechsler intelligence scales
WISC: Wechsler Intelligence Scale for Children.
